# Neuroligin 2 Is Expressed in Synapses Established by Cholinergic Cells in the Mouse Brain

**DOI:** 10.1371/journal.pone.0072450

**Published:** 2013-09-05

**Authors:** Virág T. Takács, Tamás F. Freund, Gábor Nyiri

**Affiliations:** Laboratory of Cerebral Cortex Research, Department of Cellular and Network Neurobiology, Institute of Experimental Medicine, Hungarian Academy of Sciences, Budapest, Hungary; University of Edinburgh, United Kingdom

## Abstract

Neuroligin 2 is a postsynaptic protein that plays a critical role in the maturation and proper function of GABAergic synapses. Previous studies demonstrated that deletion of neuroligin 2 impaired GABAergic synaptic transmission, whereas its overexpression caused increased inhibition, which suggest that its presence strongly influences synaptic function. Interestingly, the overexpressing transgenic mouse line showed increased anxiety-like behavior and other behavioral phenotypes, not easily explained by an otherwise strengthened GABAergic transmission. This suggested that other, non-GABAergic synapses may also express neuroligin 2. Here, we tested the presence of neuroligin 2 at synapses established by cholinergic neurons in the mouse brain using serial electron microscopic sections double labeled for neuroligin 2 and choline acetyltransferase. We found that besides GABAergic synapses, neuroligin 2 is also present in the postsynaptic membrane of cholinergic synapses in all investigated brain areas (including dorsal hippocampus, somatosensory and medial prefrontal cortices, caudate putamen, basolateral amygdala, centrolateral thalamic nucleus, medial septum, vertical- and horizontal limbs of the diagonal band of Broca, substantia innominata and ventral pallidum). In the hippocampus, the density of neuroligin 2 labeling was similar in GABAergic and cholinergic synapses. Moreover, several cholinergic contact sites that were strongly labeled with neuroligin 2 did not resemble typical synapses, suggesting that cholinergic axons form more synaptic connections than it was recognized previously. We showed that cholinergic cells themselves also express neuroligin 2 in a subset of their input synapses. These data indicate that mutations in human neuroligin 2 gene and genetic manipulations of neuroligin 2 levels in rodents will potentially cause alterations in the cholinergic system as well, which may also have a profound effect on the functional properties of brain circuits and behavior.

## Introduction

Neuroligins (NLGNs) are a family of postsynaptic transmembrane proteins that bind to presynaptic neurexins [1], whereby they form a trans-synaptic signal transduction complex and mediate a bidirectional signaling between the presynaptic axon and the postsynaptic target [2]. Both NLGNs and neurexins recruit proteins that are involved in synaptic communication and are able to induce pre- or postsynaptic specializations [3]–[5]. Experiments with NLGN-knockout (KO) mice demonstrated that NLGNs play an important role in the maturation and proper function of synapses [6], [7] and appear to be dynamically regulated and therefore contribute to the activity dependent stabilization/destabilization of synapses [8]–[11].

Four neuroligin isoforms (NLGN1-4) were described in rodent brain, which were shown to localize to different synapse types. NLGN1 is present in glutamatergic synapses [12], whereas NLGN2 was localized to GABAergic and a small subset of glycinergic synapses [4], [13], [14]. NLGN3 was found in undefined subgroups of both glutamatergic and GABAergic synaptic contacts [15]; whereas NLGN4 was detected in glycinergic synapses [16]. Consistent with the location of different isoforms, manipulation (deletion or overexpression) of NLGN1 or NLGN2 resulted in alterations in glutamatergic or GABAergic transmission, respectively [17]. The distinct localization of these NLGN isoforms suggests that they fulfill different roles in distinct synapse types and may recruit different kinds of synaptic proteins.

NLGN2 was detected exclusively in inhibitory synapses so far [4], [13], [14] and it is of particular interest, because mutations in human NLGN2 gene were implicated in schizophrenia [18], whereas manipulations of mouse NLGN2 levels resulted in characteristic behavioral phenotypes, including an increase in anxiety levels both in NLGN2-KO and NLGN2-overexpressing mice [19]–[21]. Consistent with the location of NLGN2 in inhibitory synapses, NLGN2-KO mice had impairments in inhibitory synaptic transmission [20], [22]–[24], whereas NLGN2-overexpressing animals revealed an increase in inhibition [19]. Interestingly, despite the opposite changes in the strength of GABAergic transmission detected in NLGN2-KO and NLGN2-overexpressing mice, both mice showed increased anxiety-like behavior [19], [20]. This enhancement is surprising in case of NLGN2-overexpressing mouse (where the GABAergic transmission is enhanced), because positive modulation of GABAergic signaling (for example benzodiazepine treatment) generally results in anxiolytic effects [25]. Some other behavioral and physiological effects of NLGN2-overexpression are also inconsistent with the strengthened GABAergic transmission (high level of basal activity, enhanced startle response, stereotyped jumping behavior and seizures in frontoparietal EEG [19]). These controversial results raise the possibility that besides GABAergic synapses, NLGN2 is expressed in other kinds of synapses as well. To the best of our knowledge, colocalization of NLGN2 was investigated only with glutamatergic, GABAergic and glycinergic markers, while synapses that use other types of neurotransmitters were not analyzed previously. One of the most abundant terminal type of the mammalian brain is cholinergic, and they provide a massive innervation in most brain regions [26]. They were shown to modulate almost every process in the central nervous system including development, arousal, consciousness, attention, learning and memory, anxiety and depression [27] and interestingly, in line with our hypothesis, in human, nicotine dependence was associated with neurexin-1 gene (which is one of the main binding partners of NLGNs) [28], [29].

Therefore, we tested the presence of NLGN2 in cholinergic synapses of the mouse brain using serial electron microscopic sections double labeled for NLGN2 and choline acetyltransferase (ChAT), the synthesizing enzyme of acetylcholine in axon terminals. We found that NLGN2 is expressed postsynaptically at these synapses in all investigated brain areas, and for instance in the hippocampus, its density was similar to that of the GABAergic synapses. Moreover, we also found that NLGN2 was present in atypical contact sites of cholinergic axons that probably would not have been considered contact site before, suggesting that these terminals establish more synapses than it was recognized previously. In addition, we found that cholinergic cells themselves also express NLGN2 in some of their input synapses. These results provide the basis for new interpretations of data in the literature, in which the effects of the genetic manipulation of NLGN2 was tested.

## Materials and Methods

### Ethics statement

All experiments were performed in accordance with the Institutional Ethical Codex and the Hungarian Act of Animal Care and Experimentation guidelines, which are in concert with the European Communities Council Directive of November 24, 1986 (86/609/EEC). The Animal Care and Experimentation Committee of the Institute of Experimental Medicine of Hungarian Academy of Sciences and the Animal Health and Food Control Station, Budapest, have specifically approved the experimental design under the number of 22.1/362/3/2011.

### Tissue preparation

Five male wild-type (WT) C57BL/6J mice (24–60 days old) and two neuroligin 2 knockout mice (NLGN2-KO; 49 and 67 days old) [6] were sacrificed. For perfusion, mice were anaesthetized with isoflurane followed by an intraperitoneal injection of an anesthetic mixture (containing 0.83% ketamine, 0.17% xylazin hydrochloride, 0.083% promethazinium chloride, 0.00083% benzethonium chloride, and 0.00067% hydrochinonum) to achieve deep anesthesia.

Mice were perfused transcardially with 0.9% NaCl solution for 2 min followed by a fixative containing 4% paraformaldehyde in 0.1 M phosphate buffer (PB, pH 7.4) for 35 min. In case of one WT mouse the fixative also contained 0.5% glutaraldehyde. The perfusion with fixative was followed by perfusion with PB for 10 min. The brains were then removed from the skull and coronal sections were cut on a Leica VT1200S vibratome at 50 or 60 μm. The sections were rinsed in PB, cryoprotected sequentially in 10% and 30% sucrose dissolved in PB, freezed over liquid nitrogen and stored at –70C until further processing.

### Immunohistochemistry

Sections were freeze-thawed two times over liquid nitrogen in 30% sucrose dissolved in PB. After extensive washes in PB and 0.05 M Tris-buffered saline (TBS, pH 7.4) endogenous peroxidase-like activity was blocked by incubation of the sections in 1% hydrogen peroxide in TBS for 10 min. After repeated washes in TBS, sections were blocked in 1% human serum albumin (HSA, Sigma-Aldrich, in TBS) for 1 h. This was followed by a 2–3 days of incubation in a mixture of primary antibodies for choline acetyltransferase (ChAT; monoclonal mouse antibody, 1∶750) [30] and for neuroligin 2 (NLGN2; polyclonal rabbit antibody, Synaptic Systems, Cat. No.: 129 203; Lot No. 10: 1∶600, Lot No. 12–13: 1∶300) made up in TBS containing 0.05% sodium azide. After extensive washes in TBS, sections were treated with blocking solution (Gel-BS) containing 0.2% cold water fish skin gelatin and 0.5% HSA in TBS for 1 h. This was followed by an overnight incubation in a mixture of biotinylated donkey anti-mouse antibodies (1∶1000, Jackson ImmunoResearch Europe Ltd) and 1.4-nm gold-conjugated goat anti-rabbit antibodies (1∶100–300; Fab' fragment, Nanoprobes) diluted in Gel-BS. After repeated washes in TBS and PB, sections were treated with 2% glutaraldehyde in PB for 15 min to fix the gold particles into the tissue. This was followed by washes in PB, TBS, and a 2–3 hours of incubation in Elite ABC (1∶300, Vector Laboratories) diluted in TBS. After sections were washed in TBS and tris-buffer (pH 7.6) the immunoperoxidase reaction was developed using 3,3-diaminobenzidine (DAB) as chromogen. After repeated washes in PB and Enhancement Conditioning Solution (Aurion), gold particles were intensified using the Aurion R-Gent Silver Enhancement Solution (SE-EM) as described by the manufacturer. After subsequent washes in PB, sections were treated with 0.5% osmium tetroxide in PB for 8–15 min on ice, dehydrated in ascending ethanol series and acetonitrile and embedded in epoxy resin (Durcupan, ACM, Fluka). During dehydration sections were treated with 1% uranyl acetate in 70% ethanol for 20 min.

### Electron microscopy

For electron-microscopic analysis of cholinergic terminals, resin-embedded tissue samples from the CA1 area of the dorsal hippocampus, caudate putamen (CPu), basolateral amygdala (BLA), centrolateral thalamic nucleus (CL), somatosensory (S1) and medial prefrontal cortices (PFC) were glued onto small Durcupan blocks. Series of consecutive ultrathin sections (70 nm thick, at least 14 sections/series) were cut using an ultramicrotome (Leica EM UC6) and picked up on Formvar-coated single-slot grids. Ultrathin sections were counterstained with lead citrate (Ultrostain 2, Leica) and examined in a Hitachi 7100 electron microscope equipped with a Veleta CCD camera (Olympus Soft Imaging Solutions, Germany). For evaluation of the NLGN2 content at synapses of ChAT-positive terminals, sections were systemically scanned for synapses of DAB-labeled ChAT-positive boutons. Parallel appositions between the membranes of the presynaptic bouton and the putative postsynaptic target were regarded as synapses if they displayed widening of the extracellular space at the presumptive synaptic cleft, a postsynaptic membrane thickening, and clustered synaptic vesicles in the bouton. Synapses found were followed and photographed at 30,000 magnification in every section where they were present throughout the series: thus these synapses were fully reconstructed. For the semiquantitative analyses, we measured the length of synapses from these series of digital images using the ImageJ image analyzer software (NIH, USA) then counted the immunogold particles at the postsynaptic membrane. Gold particles were considered to be associated with the cell membrane only when they were not farther away from the membrane than 40 nm. The density of immunogold particles at extrasynaptic plasma membranes and type I synaptic membranes of the target profiles was also measured.

For comparison of NLGN2 contents of ChAT-positive and GABAergic terminals in the hippocampus, we have also measured the immunogold densities of partially or fully reconstructed somatic synapses in the pyramidal layer of the hippocampal CA1 area, because hippocampal pyramidal cells receive only GABAergic synapses onto their somata in rodents [31]. These synapses were reconstructed from the very same series of sections.

Postsynaptic targets of hippocampal cholinergic terminals were classified as described earlier [32]. Briefly, spines were recognized by their small size and specific morphology. Dendrites that have spines and do not receive type I (asymmetric) inputs on their shafts are known to be pyramidal cells [31], whereas dendrites receiving type I synapses on their shafts are interneurons [33]. The robustness of this classification method was reconfirmed recently [32]. Cell bodies from str. pyramidale that did not receive type I inputs were considered to be pyramidal cells, whereas cell bodies in other layers were classified as interneurons. In other brain areas (see above) only the dendrites and spines were discriminated.

For electron microscopic analysis of input synapses of cholinergic cells, tissue samples were taken from medial septum (MS), vertical- and horizontal limbs of the diagonal band of Broca (VDB and HDB), substantia innominata/ventral pallidum (SI/VP) and CPu. Consecutive series of ultrathin sections were systematically scanned for NLGN2-positive synapses of DAB-labeled ChAT-positive dendrites and somata. MS, VDB, HDB and SI/VP were also scanned for NLGN2-positive synapses of ChAT-positive terminals.

### Specificity of antibodies

**Figure 1:**
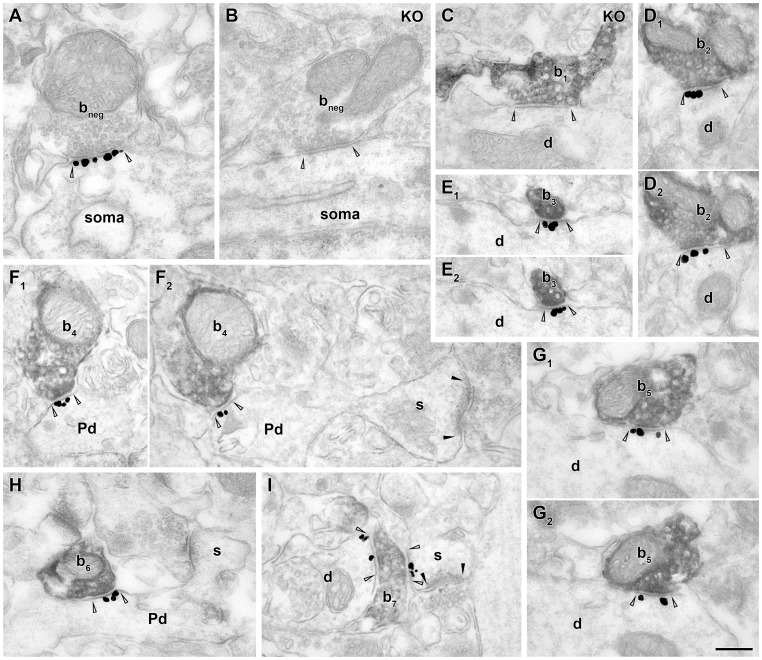
Neuroligin 2 is present postsynaptically at both GABAergic and cholinergic synapses in the hippocampus. Electron micrographs from combined immunogold/immunoperoxidase experiments for NLGN2 (immunogold: black particles) and ChAT (DAB: dark, homogenous reaction product) reveal the presence of NLGN2 at ChAT-negative and ChAT-positive type II synapses in the CA1 area. Arrowheads indicate synapse-edges. A, A pyramidal cell body receives a synapse from a ChAT-negative bouton (b_neg_) that expresses NLGN2 postsynaptically in a WT mouse. B, C, In contrast, the same type of immunostaining in a NLGN2-KO mice shows no NLGN2-immunoreactive synapses, demonstrating the specificity of the antibody. A GABAergic terminal (b_neg_) from str. pyramidale, lacking gold particles at the postsynaptic site is shown (B). An example of a synapse of a ChAT-positive bouton (b_1_) on a dendrite (d) in str. radiatum that is immunonegative for NLGN2 in KO mouse (C). D–I: NLGN2 immunogold labeling is present at the postsynaptic site of synapses established by ChAT-positive axon terminals (b_2–5_) on dendrites (d) and spines (s) in str. radiatum (D–G) and oriens (H, I) of WT mice. Serial images show the same synapse in D_1_ and D_2_; E_1_ and E_2_; F_1_ and F_2_; G_1_ and G_2_. E_1–2_ demonstrates that some of the presynaptic profiles were small-diameter, intervaricose-like segments of ChAT-positive axons (b_3_). In F_1–2_ and H, the postsynaptic targets of boutons b_4_ and b_6_ are putative pyramidal dendrites (Pd) the latter of which is identified by the presence of spines (s). I, Occasionally, we found ChAT-positive presynaptic elements that formed synapses with two postsynaptic targets. Here, bouton b7 forms a synapse with a dendrite and a spine, which receives a type I synapse (black arrowheads). Note, that in many cases, synaptic junctions of ChAT-positive terminals are atypical (E, F, H, I). Scale bar is 200 nm for all images.

We tested the NLGN2 antibody in experiments with NLGN2-KO mice (n = 2). At the electron microscopic level, specific labeling of synapses could not be detected in these animals (Fig. 1B and C). We have also investigated 28 completely reconstructed synapses of hippocampal ChAT-positive terminals from two NLGN2-KO mice and found only one gold particle in only one synapse. Therefore, the density of synaptic labeling in WT animals was 240-fold larger than in NLGN2-KO mice (9.6±6.02 vs. 0.04±0.24 intensified gold particles/ µm) demonstrating that the background labeling is negligible. The ChAT antibody was used in several previous studies [34]–[39], and its specificity has been characterized previously [30].

### Statistical Analysis

A statistical analysis was carried out using the software Statistica (StatSoft). When data populations had a Gaussian distribution according to the Shapiro-Wilk's W test, we reported parametric statistical features (mean ± SD). In the case of non-Gaussian distribution, we used non-parametric statistical features (median, interquartile ranges). Two groups showing Gaussian distribution were compared using the parametric t test. The Kruskal-Wallis test was used to compare the data from three groups showing non-Gaussian distribution. The differences were considered significant at p< 0.05.

## Results

### Neuroligin 2 is abundant at hippocampal cholinergic synapses

Although NLGN2 is widely considered to be present only in GABAergic synapses [2], [7], [40]–[44], we tested its presence at cholinergic synapses as well. We performed double immunogold/ immunoperoxidase labeling for NLGN2 and choline acetyltransferase (ChAT), the synthesizing enzyme of acetylcholine. In the hippocampus of NLGN2-KO mice, no specific NLGN2 labeling was found (see Methods, Fig. 1B and C). First, we tested the presence of NLGN2 in GABAergic synapses. CA1 pyramidal cells were shown to receive exclusively GABAergic synapses onto their somata in rodents [31], therefore these synapses were considered to be GABAergic. We confirmed the presence of NLGN2 in these type II (symmetric) synapses of GABAergic boutons (Fig. 1A) [13], [45].

Interestingly, synapses of ChAT-positive terminals were also densely labeled at the postsynaptic membrane (Fig. 1D–G). To estimate and compare the abundance of NLGN2 in cholinergic and GABAergic synapses we tested fully reconstructed synapses of ChAT-positive terminals from str. radiatum (n = 59), pyramidale (n = 13) and oriens (n = 35) and fully or partially reconstructed synapses of GABAergic somatic boutons (n = 69) on pyramidal cell bodies in the CA1 area of three mice. Hippocampal cholinergic boutons formed type II synapses that were usually very small (they were present typically only in 2–4 (2.9±1.2) 70 nm-thick sections, median of synaptic membrane area: 0.0256 μm^2^, interquartile range: 0.0205–0.0369 μm^2^; n = 107, three mice, pooled, Fig. 1D–I) compared to GABAergic synapses. For example, the size of parvalbumin and cannabinoid receptor 1 positive somatic synapses per contact are about 0.07 and 0.22 μm^2^, respectively (our unpublished observations). Please note, that although synapses were collected in a random fashion, these are only semiquantitative measurements, nevertheless they still clearly demonstrate the tendency that cholinergic synapses are smaller than GABAergic ones.

**Figure 2:**
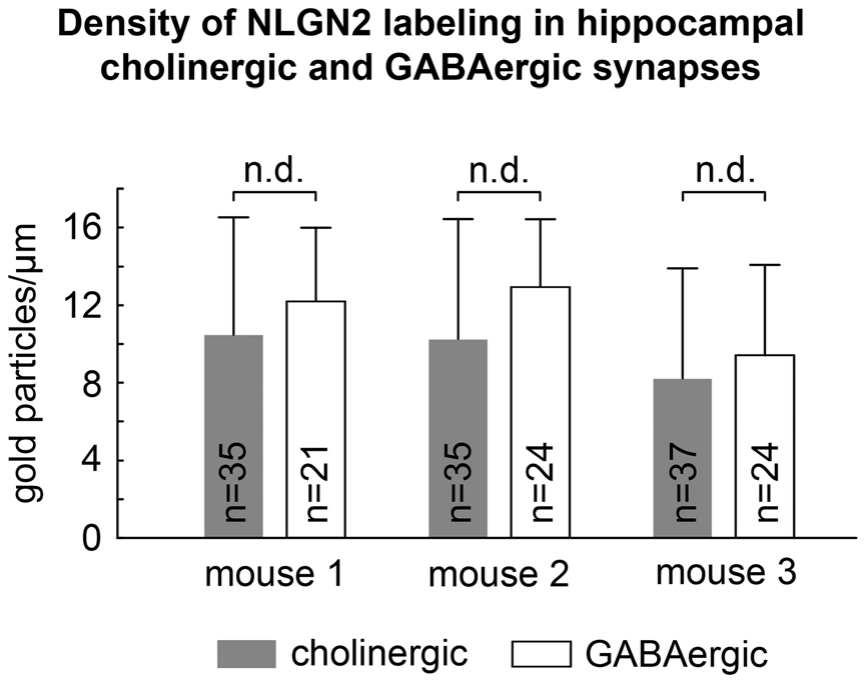
Hippocampal cholinergic and GABAergic synapses express a similar density of neuroligin 2. There was no significant difference in the density of NLGN2 labeling (intensified gold particles/ µm synaptic membrane) between GABAergic synapses on pyramidal cell somata (12.2±3.8, 13±3.5 and 9.4±4.7; mean and standard deviation in three mice, respectively) and ChAT-positive synapses in str. radiatum, pyramidale and oriens (10.5±6.1, 10.2±6.2 and 8.2±5.7). (n.d.: no statistical difference).

In three WT mice, 100%; 100% and 95.8% of the GABAergic synaptic connections (n = 68 out of 69) and 94.3%; 97.1% and 86.5% of cholinergic synapses (n = 99 out of 107) were identified as NLGN2 positive on the basis of intensified immunogold particles associated with the postsynaptic membrane. The somewhat lower positivity of the cholinergic synapses may be due to the fact that they could be tested on fewer sections, because they are much smaller (see above). To test the relative density of NLGN2 in these synapses and extrasynaptically as well, we measured and calculated the relative density of the immunogold labeling. The labeling was specifically enriched in GABAergic and cholinergic synapses compared to the labeling in extrasynaptic membranes and type I synapses (for the definition of membrane associated immunogold particles, please see methods). In three mice, the linear density of labeling was 12.2±3.8; 13±3.5 and 9.4±4.7 gold particles per μm membrane (mean ± SD) in GABAergic synapses, whereas it was only 0.11±0.1; 0.12±0.06 and 0.06±0.06 gold particles per μm at extrasynaptic membrane domains of the same somata in the vicinity of these synapses. In the same animals, in cholinergic synapses, the linear density of labeling was 10.5±6.1; 10.2±6.2 and 8.2±5.7 gold particles per μm membrane, whereas it was only 0.11±0.15; 0.1±0.11 and 0.13±0.19 gold particles per μm at extrasynaptic and type I synaptic membranes of the postsynaptic targets of cholinergic boutons. The linear density values of NLGN2 labeling at GABAergic and cholinergic synapses were compared in three mice and no significant differences were found (Fig. 2). We identified the postsynaptic targets of cholinergic boutons in three mice, and found that at least 48.8%; 68.6% and 48.6% of them innervated pyramidal dendritic shafts (Fig. 1F and H) and 17.1%; 20%; and 24.3% targeted spines, that also received a type I input (Fig. 1I). Only 2.9%; 0% and 8.1% of the cholinergic synapses targeted interneuron dendrites or somata (three interneuron dendrites and one interneuron soma out of 107 targets), and rarely cholinergic boutons innervated pyramidal cell somata as well (two out of 107 targets; 0%; 2.9% and 2.7% of the boutons in three mice). The rest of the postsynaptic targets could not be unequivocally classified (31.4%; 8.6% and 16.2%). Occasionally, we found cholinergic boutons that formed two synapses with different postsynaptic targets (Fig. 1I).

These data show that in the hippocampus virtually all cholinergic synapses contain NLGN2 at the postsynaptic membrane and its density is just as high in cholinergic as in GABAergic synapses.

### Neuroligin 2 is also abundant in neocortical cholinergic synapses

**Figure 3:**
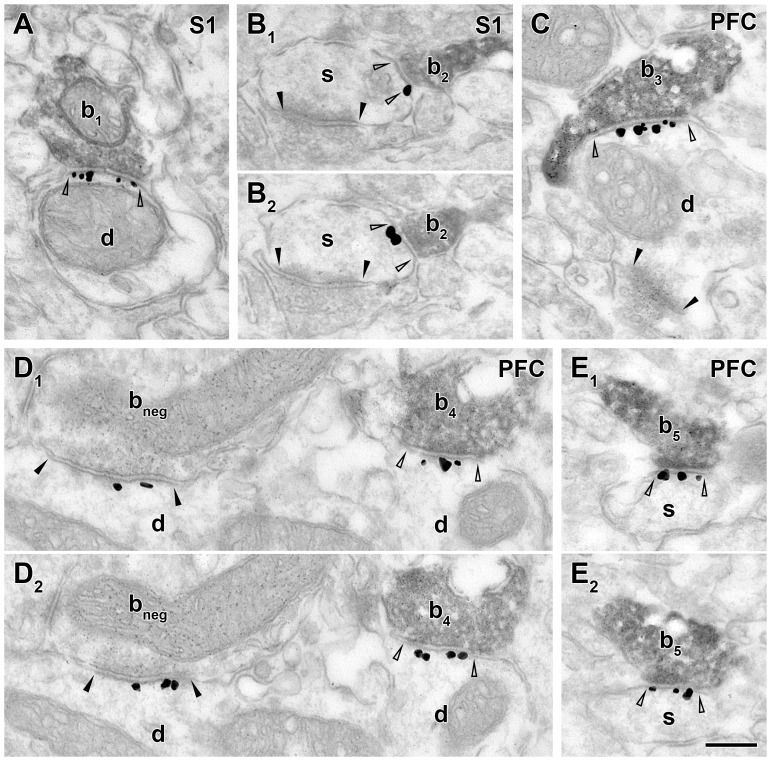
Neuroligin 2 is localized postsynaptically at cholinergic synapses in the neocortex. Images demonstrate double immunohistochemical reactions for ChAT (dark, homogenous DAB precipitate) combined with NLGN2 (black intensified gold particles) in somatosensory (S1) and prefrontal cortices (PFC). Serial sections of the same synapses are shown in B_1–2_, D_1–2_ and E_1–2_. In both areas, ChAT-positive boutons (b_1–5_) form type II synaptic contacts on dendrites (d, A, C, D_1–2_) and spines (s, B_1–2_, E_1–2_) that express NLGN2 at the postsynaptic membranes (open arrowheads label synaptic edges). The innervated spines also received a type I synapse from a ChAT-negative terminal (B_1–2_, black arrowheads). In C the postsynaptic dendrite of bouton b_3_ receive an additional, type I synaptic input (black arrowheads) from an unlabeled terminal. These type I synapses in B_1–2_ and C do not contain NLGN2. In contrast, another ChAT-negative, putative GABAergic bouton (b_neg_) establishes a type II, NLGN2-positive synapse (black arrowheads) with a dendrite in D_1–2_. Scale bar is 200 nm for all images.

We tested the presence of NLGN2 in the somatosensory (S1) and prefrontal cortices (PFC) using the same combined immunogold/ immunoperoxidase staining for NLGN2 and ChAT as above. In the S1 of two WT mouse, 97% and 88.6% of the cholinergic synapses (n = 33, 35) were identified as NLGN2 positive on the basis of intensified immunogold particles associated with the postsynaptic membrane (Fig. 3A, B). Cholinergic terminals formed small type II synapses on dendritic shafts (60.6% and 60% of all targets; Fig. 3A) and spines (36.4% and 37.1%; Fig. 3B) in the S1, whereas 3% and 2.9% of the postsynaptic targets remained unidentified. Many of the innervated dendritic shafts possessed spines on the recorded serial photos (21.2% and 37.1% of all targets), demonstrating that these originated presumably from pyramidal cells. In the PFC, almost all cholinergic synapses collected were NLGN2-positive (92% and 84.8%; n = 25 and 46, two mice; Fig. 3C–E). The morphology and size of synapses were also similar in these cortical areas: they formed type II synapses that were usually small. The distribution of postsynaptic targets were also similar in the S1 and PFC: in the PFC of two mice 64% and 41.3% of the cholinergic synapses were found on dendrites (Fig. 3C, D; 20% and 8.7% of all targets were on putative pyramidal dendrites, because they were spiny on the serial photos), whereas 24% and 52.2% of the cholinergic inputs innervated spines (Fig. 3E). Some (12% and 6.5%) of the targets could not be classified.

### Neuroligin 2 is expressed in cholinergic synapses in several other non-cortical brain areas as well

The basolateral amygdala (BLA), the caudate putamen (CPu) and the thalamic centrolateral nucleus (CL) receive abundant cholinergic innervation [46].

**Figure 4:**
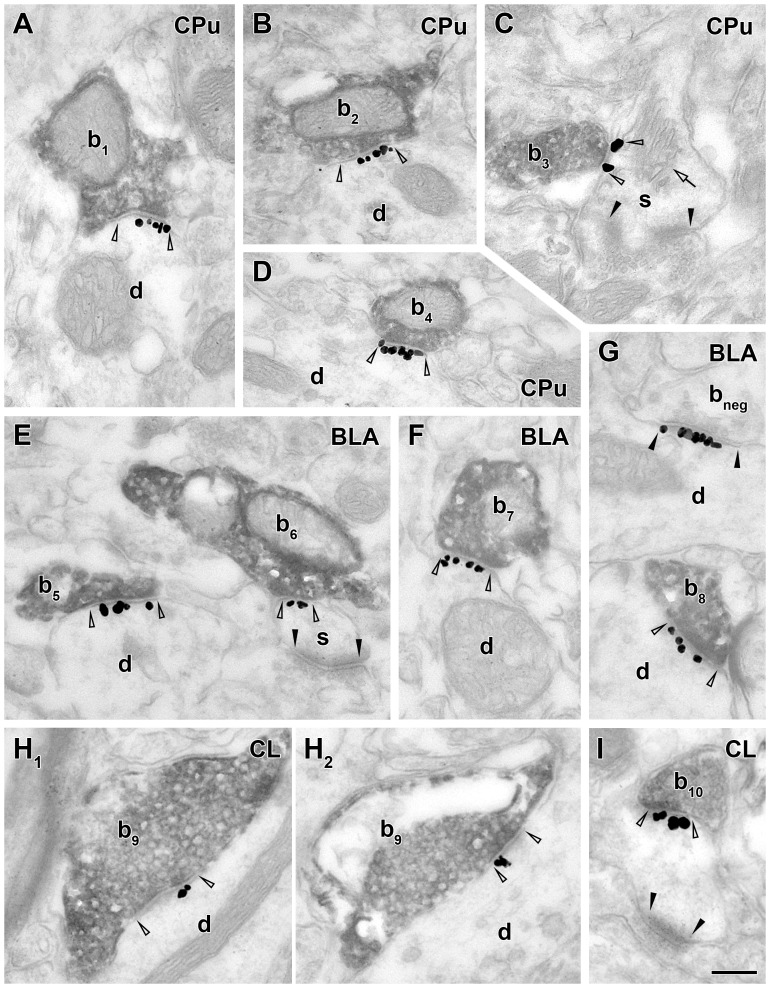
Neuroligin 2 is expressed postsynaptically at cholinergic synapses in the caudate putamen, basolateral amygdala and centrolateral thalamic nucleus. Electron micrographs from combined immunogold/immunoperoxidase experiments show that NLGN2 immunogold labeling (black particles) is present at the postsynaptic site of synapses (open arrowheads) established by ChAT-positive axon terminals (b_1–10_, dark reaction product) on dendrites (d) and spines (s) in caudate putamen (CPu, A–D) basolateral amygdala (BLA, E–G), and centrolateral thalamic nucleus (CL, H–I). Note that many of these boutons form synapses that could hardly be identified without NLGN2 labeling (e.g. b_3_ in C, b_6_ in E or b_8_ in G). In C, arrow indicates the spine apparatus. In G, a putative GABAergic bouton (b_neg_) forming a NLGN2-positive type II synapse (black arrowheads) is also shown next to the ChAT-positive terminal. Serial sections of the same terminal are shown in H_1–2_. Scale bar is 200 nm for all images.

In BLA, cholinergic terminals formed type II synapses that were NLGN2-positive (97.1% and 100%; n = 34 and 47, two mice; Fig. 4E–G). Cholinergic boutons innervated dendrites (76.5 and 51.1% of all targets in two mice; Fig. 4E–G) and spines (17.6% and 48.9%; Fig. 4E); 2.9 and 0% of the targets could not be classified. One of the postsynaptic targets (out of 81) was an unidentified soma. Large part of the dendritic targets possessed spines in the examined segment (35.3% and 40.4% of all targets), suggesting that they originated from pyramidal cells.

In CPu, cholinergic synapses formed type II synapses that were NLGN2-positive (92% and 97.6% n = 25 and 41, two mice; Fig. 4A–D). Approximately every second cholinergic synapse innervated dendrites in CPu (48% and 43.9% of all targets; Fig. 4A,B and D) whereas the rest of them targeted spines (36% and 36.6%; Fig. 4C); 16% and 14.6% of all targets remained unidentified. Out of 66 synapses tested, one targeted a soma and another targeted an axon initial segment. 36% and 9.8% of all targets were spiny dendrites, suggesting that they were GABAergic medium spiny neurons in CPu.

In contrast to other brain areas investigated, cholinergic synapses formed both type I and type II synapses in CL (53.3% type I, 30% type II in the first animal; 16% type I, 56% type II in the second animal; the remaining synapses could not be classified; n = 30 and 25, two mice). Most cholinergic synapses were NLGN2-positive in CL (73% and 84%; Fig. 4H, I).

We also tested the NLGN2 content of ChAT-positive synapses in basal forebrain areas and found that their cholinergic terminals formed NLGN2-positive type II synapses. The number of positive samples that were collected are as follows: the medial septum: n = 14 and 2; vertical limb of diagonal band of Broca: n = 3 and 7; horizontal limb of diagonal band of Broca: n = 4 and 10; substantia innominata/ ventral pallidum: n = 9 and 12 synapses collected from two mice, respectively).

### Neuroligin 2 clusters revealed the presence of contact sites of cholinergic boutons that probably would not have been considered contact sites previously

In our preliminary experiments, we expected cholinergic terminals to establish synapses only rarely in cortical areas [34], [35], [38], [39]. Although cholinergic synapses are known to be less prominent than GABAergic or glutamatergic synapses, we expected and searched for typical synapse features. Indeed, several cholinergic synapses had typical synaptic morphology and NLGN2 labeling. Based on data in the literature and on our own experience, NLGN2 is accumulated only in synaptic contact sites and indeed its clusters appeared in synapses with very typical morphology. However, it very soon became obvious that not all cholinergic contact sites resemble typical features of type I or II synapses. In several cases, we found clusters of NLGN2 immunogold labeling next to ChAT positive terminals, in membrane appositions that traditionally would not have been considered synaptic contact sites previously, because of an only very mild thickening of the membrane and because its size is smaller than the smallest cortical GABAergic synapses. However, based on the accumulated data, these contact sites should probably also be considered synapses.

Especially in the hippocampus and neocortex, a substantial amount of the cholinergic contact sites possessed hardly detectable thickening of the synaptic membranes (Fig. 1E, F, H, I; Fig. 3B, E). Some of these contacts were formed by small-diameter intervaricose segments of cholinergic axons (Fig. 1E). In many cases, the length of parallel appositions between membrane segments of the presumed pre- and postsynaptic profiles were also small; therefore probably none of these contact sites could have been recognized without NLGN2 labeling. However, these contacts were as densely labeled for NLGN2 as cholinergic synapses that have more prominent synaptic clefts and postsynaptic densities (Fig. 1.D, 3A, C). According to our observations, there is a continuum between clearly apparent synapse-like structures of cholinergic boutons and hardly detectable contact sites that can now be recognized based on the dense NLGN2 labeling.

### Cholinergic cells themselves express neuroligin 2 in a subset of their input synapses

**Figure 5:**
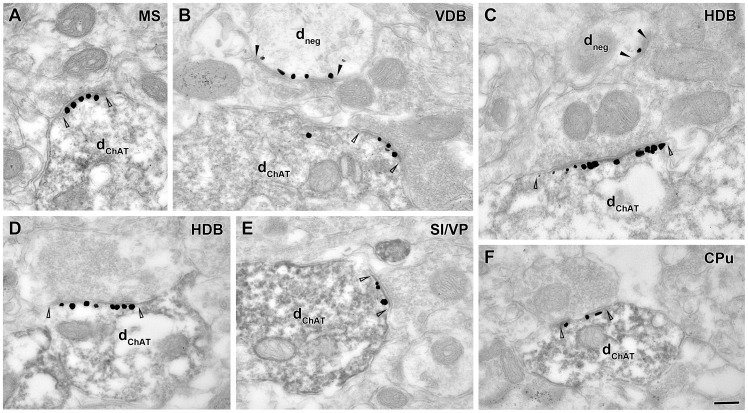
Cholinergic projection neurons of the basal forebrain and neostriatal cholinergic interneurons express NLGN2 in their inputs synapses. Images from combined immunogold/immunoperoxidase experiments show that dendrites of cholinergic cells (dChAT, dark, homogenous DAB precipitate) express NLGN2 (intensified gold particles) at postsynaptic membranes of type II synapses (open arrowheads) in the medial septum (A: MS), vertical- and horizontal diagonal band of Broca (B: VDB; C, D: HDB), substantia innominata/ventral pallidum (E: SI/VP) and caudate putamen (F: CPu). In B and C two unlabeled dendrites (d_neg_) also express NLGN2 in their type II synapses (black arrowheads). Scale bar is 200 nm for all images.

The majority of cholinergic afferents to most of the investigated brain areas (hippocampus, BLA, S1, PFC) arises from different parts of the basal forebrain: the medial septum (MS), the vertical and horizontal limbs of the diagonal band of Broca (VDB and HDB) and the substantia innominata/ ventral pallidum (SI/VP) [26]. We tested whether these cholinergic cells of the basal forebrain express NLGN2 at their input synapses on their dendrites and/or cell bodies. We found that a subset of their synaptic inputs were indeed NLGN2-positive (Fig. 5A–E). NLGN2-positive input synapses of cholinergic cells were collected in all of the basal forebrain areas investigated (MS: n = 24 dendritic and n = 24 somatic; VDB: n = 29 dendritic and n = 11 somatic, HDB: n = 37 dendritic and n = 14 somatic, SI/VP: n = 50 dendritic and n = 10 somatic synapses from two mice). In the MS, two of the somatic, and one of the dendritic NLGN2-positive input synapses were formed by ChAT-positive terminals, while one similar dendritic input was found in SI/VP, demonstrating that cholinergic cells can form synaptic connections with each other and these contacts also contain NLGN2.

In contrast to most of the brain areas that receive their cholinergic innervation from distant projection neurons, dense cholinergic innervation in CPu is provided by local cholinergic interneurons [47]. We found that they also express NLGN2 in a minority of their input synapses (n = 43 dendritic and n = 7 somatic NLGN2-positive synapses were collected from two mice; Fig. 5F).

## Discussion

The present study provides evidence that besides GABAergic synapses, cholinergic synapses also express NLGN2 postsynaptically, in all investigated brain areas in mice. Our estimation also shows that NLGN2 density is similar in cholinergic and GABAergic synapses in the hippocampus. We identified several putative contact sites established by cholinergic axons that do not show the typical morphology of classical synapses and therefore, they probably could not be recognized without NLGN2 labeling. We also found that some of the input synapses of cholinergic cells contain NLGN2 postsynaptically, demonstrating that cholinergic cells themselves also express NLGN2.

NLGNs are present postsynaptically and form a trans-synaptic signal transduction complex with presynaptic neurexins; they participate in the recruitment of synaptic proteins, and thereby play an important role in the maturation and activity-dependent regulation of synaptic contacts [3], [4], [6], [9]–[11]. Previous immunocytochemical experiments localized NLGN2 exclusively to GABAergic and a small subset of glycinergic synapses [13], [14]. Further investigations and conclusions were based on those results in the literature and several studies demonstrated that deletion of NLGN2 caused selective impairment in inhibitory synaptic transmission [17], [22]–[24], whereas overexpression of NLGN2 resulted in enhanced inhibition [17], [19]. However, in the light of our results, previous conclusions may need to be reconsidered, because the strength of cholinergic synapses was probably also altered in NLGN2-KO and NLGN2-overexpressing animals.

### Potential molecular interactions of neuroligin 2 in cholinergic synapses

In perisomatic GABAergic synapses, NLGN2 was shown to bind to the GABA_A_-receptor anchoring protein gephyrin and to activate collybistin, which is responsible for the membrane tethering of gephyrin [23]. Through this interaction, NLGN2 participates in the clustering of GABA_A_ receptors at the postsynaptic side, which likely influences the properties of GABAergic synapses [23], [24], [48]. Cholinergic synapses are much less known than GABAergic ones, however; here, NLGN2 may also contribute to the recruitment or alignment of synaptic proteins, including acetylcholine (ACh) receptors in cholinergic synapses.

What kind of scaffolding proteins can potentially bind to NLGN2 in cholinergic synapses? The synaptic scaffolding molecule (S-SCAM; [49]) is also present and interacts with NLGN2 at inhibitory synapses [50]. Furthermore, it was demonstrated that in chicken, where only three forms of NLGN is present (NLGN1,3,4) and NLGN2 is absent [51], S-SCAM is directly associated with NLGN1 at cholinergic synapses of the ciliary ganglion [52]. In these synapses, S-SCAM indirectly interacts with the adenomatous polyposis coli protein that organizes a multimolecular protein complex which targets α3 nicotinic ACh receptors to the postsynaptic membrane [53], [54]. With these interactions NLGN1 might indirectly influence the strength of these cholinergic contacts. NLGN1 also binds to β-neurexins in these cholinergic synapses, induces accumulation of presynaptic components and enhances nicotinic synaptic activity in chicken ciliary ganglionic cell culture [55]. A third known binding partner of NLGN1 in cholinergic synapses of the chicken ciliary ganglion is the scaffolding protein postsynaptic density-93 [52], which is also present in cholinergic synapses of autonomic ganglia in mouse and plays a role in stabilization of nicotinic ACh receptors at postsynaptic sites [56]. Although, to the best of our knowledge, no data is available about the presence of these three proteins in cholinergic synapses of the mammalian central nervous system, but our results suggest that they may potentially be present and interact with NLGN2 in these synapses.

It is known that different NLGN isoforms can be present in the same synapse. For instance, a subset of GABAergic synapses express both NLGN 2 and 3; while several glutamatergic synapses express both NLGN 1 and 3 [15]. Therefore, cholinergic synapses may also express other types of NLGNs. However, it is not possible to predict, whether other neuroligin isoforms are also present at these synapses, because (besides heterodimers [57]) NLGNs can also form homodimers by themselves.

### Possible role of neuroligin 2 in cholinergic synapses

The behavioral phenotype of NLGN2-KO mice and NLGN2-overexpressing animals has been thoroughly described [19]–[21], [58]. However, the interpretation of these data should be reconsidered in the light of our new results.

NLGN2-KO mice showed a marked increase in anxiety-like behavior which can be explained by an impairment in GABAergic synaptic transmission [20], because pharmacological blockade of GABA_A_ receptors produces a similar effect [25], [59]. In contrast, positive modulators of GABAergic signaling cause anxiolysis [25], [59], [60]. Based on these data, the expected effect of NLGN2 overexpression in GABAergic synapses would be also anxiolytic. However, global overexpression of NLGN2 resulted in an enhancement of anxiety-like behavior despite the observed potentiated GABAergic function [19]. We suggest that these controversial findings may be explained by a strengthened cholinergic tone that is due to NLGN2-overexpression, the result of which may be a subsequent strengthening of these synapses. This is supported by findings that increased ACh signaling can indeed induce anxiety-like behavior [61]–[63]. Nevertheless, it should also be noted that manipulations of the cholinergic system, for example nicotine administration, can also induce anxiolytic effects, depending on treatment type, dose and brain region investigated [64].

In NLGN2-overexpressing mice, high levels of basal activity, enhanced startle response, anxiety and stereotyped jumping behavior was observed [19] that may be the result of an increased level of arousal. We have detected NLGN2 postsynaptically at virtually all cholinergic synapses investigated, including those in the intralaminar thalamus, a brain area that receives cholinergic innervation from the pedunculopontine nucleus [65]. This projection is part of the cholinergic arm of the reticular activating system [65], therefore, if NLGN2 content of these synapses is increased in NLGN2-overexpressing mice, their arousal state could also be heightened.

The general effect of NLGN2 manipulation may also depend on the relative number of cholinergic terminals in different brain regions. In the hippocampus, according to data published by different groups, the density of GABAergic terminals seems to be larger (1.93 x 10^8^ GAD-immunoreactive boutons/mm^3^ in CA1 area of rat [66]) than the density of cholinergic varicosities (4.9 and 5.6 x 10^6^ ChAT-positive varicosities/mm^3^ in CA1 area of rat and mouse, respectively [37]). Since the density of NLGN2 is similar in cholinergic and GABAergic synapses, but GABAergic synapses are larger and more numerous, the majority of NLGN2 is localized in GABAergic synapses in the hippocampus. However, the relative proportion of cholinergic terminals may outnumber GABAergic terminals in other brain areas. For instance, in the striatum, cholinergic terminals are highly abundant (2 x 10^8^ varicosities/mm^3^ [36]) and in these areas manipulation of NLGN2 will probably have a higher impact on the cholinergic system. Nevertheless, the importance of a molecule at the network level is not necessarily a linear function of its abundance in the given brain area.

The incidence of seizure spiking in the frontoparietal EEG of NLGN2-overexpressing mouse [19] may also be the consequence of an increased cholinergic tone, because brain oscillations are under effective cholinergic control [67], [68].

Since the cholinergic system influences also brain circuits that are responsible for regulating motor control, NLGN2 loss in cholinergic synapses might also contribute to the slightly diminished motor co-ordination described in NLGN2-KO mice [20], [21]. This hypothesis is supported by the observations that gait and postural instability might be associated with cholinergic dysfunction in Parkinson's disease as well [69].

Mutations of NLGN2 gene were recently identified in schizophrenia patients [18]. Because cholinergic impairments are implicated in schizophrenia [70], NLGN2 dysfunction in cholinergic synapses may also contribute to the pathophysiology in these subjects.

### Cholinergic and GABAergic cells express neuroligin 2 in their inputs synapses

We demonstrated that cholinergic neurons of the basal forebrain and striatum express NLGN2 in the postsynaptic membrane of their putative GABAergic (ChAT-negative, type II) and cholinergic (ChAT-positive) inputs synapses. In the hippocampus, a small fraction of the postsynaptic targets of NLGN2-positive cholinergic synapses were identified as GABAergic interneurons, which means that GABAergic cells also express NLGN2.

### Neuroligin 2 in non-classical cholinergic contacts

Interestingly, besides classical synapses of cholinergic axons, we found NLGN2 clusters also at cholinergic contact sites that did not resemble typical synapses because of their small size and a very mild thickening of the postsynaptic membrane. Because NLGN2 is known to recruit other synaptic receptor proteins, these clusters may label membrane segments that participate in signal transmission. Therefore, these contacts might be considered synapses that do not show classical morphology. Many studies that analyzed the incidence of synaptic contacts formed by cholinergic boutons concluded that cholinergic innervation of the brain is mainly non-synaptic [34]–[36], [38], [39]. These studies provided strong support for the volume transmission hypothesis [71]. In contrast, other groups demonstrated that classical synaptic contacts predominate among cholinergic inputs [72], [73]. Because we could see a continuum between clearly synapse-like contacts and those contacts that could be recognized only with the aid of NLGN2-labeling, the discrepancy between these groups of studies might be explained by a different strictness of criteria used in the morphological definition of the synaptic active zone. Because a completely different approach would be required, we did not attempt to quantify the proportion of boutons or inter-bouton (i.e. intervaricose) segments (Fig. 1E) that formed synaptic contacts in this study. Nevertheless, our results imply that cholinergic synapses are far more frequent than presumed previously, even if several of them do not show classical morphology. This may suggest the importance of synaptic transmission also in the cholinergic system. Indeed, for instance, acetylcholinesterase (that terminates the ACh signal) has a very high catalytic activity [74], [75], and it is particularly abundant in the striatum [76]. These facts suggest that ACh is quickly cleared from extracellular space; therefore, synaptic transmission of ACh would be more effective in this area. New data on phasic ACh release also support this view [75].

Previously, NLGN2 was found in GABAergic and some glycinergic synapses [13], [14], while we found it in cholinergic synapses in this study. However, information about the possible presence or absence of NLGN2 in dopaminergic, noradrenergic, serotonergic and some other types of synapses is still to be explored.
